# Costs and consequences of chronic pain due to musculoskeletal disorders from a health system perspective in Chile

**DOI:** 10.1097/PR9.0000000000000656

**Published:** 2018-09-10

**Authors:** Constanza Vargas, Norberto Bilbeny, Carlos Balmaceda, María Francisca Rodríguez, Pedro Zitko, Rubén Rojas, María Eliana Eberhard, Marisol Ahumada, Manuel Antonio Espinoza

**Affiliations:** a Health Technology Assessment Unit, Center of Clinical Research, Faculty of Medicine, Pontificia Universidad Católica de Chile, Santiago, Chile; b Business School, Centre for Health Economics Research and Evaluation (CHERE), University of Technology Sydney, Sydney, Australia; c Asociación Chilena para el Estudio del Dolor (ACHED), Santiago, Chile,; d Faculty of Medicine, Universidad San Sebastián, Santiago, Chile,; e Department of Public Health, Faculty of Medicine, Pontificia Universidad Católica de Chile, Santiago, Chile

**Keywords:** Chronic pain, Cost, Economic evaluation, Chile

## Abstract

**Background:**

Chronic pain is a prevalent and distressing condition caused by an unceasing pain lasting more than 3 months or a pain that persists beyond the normal healing time. There is evidence of inadequate management partly explained by the unawareness regarding the magnitude of the problem.

**Objectives:**

To estimate the annual expected costs and consequences of chronic pain caused by musculoskeletal diseases from the health system perspective in Chile.

**Methods:**

A Markov cohort model was built to represent chronic pain and estimate expected costs and consequences over 1-year time horizon. Transition probabilities were obtained through expert elicitation. Consequences examined were: years lost to disability (YLD), depression, anxiety, and productivity losses. Direct health care costs were estimated using local sources. Probabilistic sensitivity analysis was performed to characterize second-order uncertainty.

**Results:**

The annual expected cost due to musculoskeletal chronic pain was estimated in USD $1387.2 million, equivalent to 0.417% of the national GDP. Lower back pain and osteoarthritis of the knee explained the larger proportion of the total cost, 31.8% and 27.1%, respectively. Depression attributed to chronic pain is another important consequence accounting for USD $94 million (Bayesian credibility interval 95% $49.1–$156.26). Productivity losses were also important cost, although early retirement and presenteeism were not measured. Chronic pain causes 137,037 YLDs.

**Conclusion:**

Chronic pain is not only an important cause of disability but also responsible for high social and financial burden in Chile. Public health programs focused on managing chronic pain may decrease burden of disease and possibly reduce costs.

## 1. Introduction

Chronic pain has been defined as an unceasing pain lasting more than 3 months or a pain that persists beyond the normal healing time.^18^ It is a multifactorial condition that manifests with physical and psychological symptoms causing multiple consequences, including the decrease in quality of life, changes in mood, impaired function, sleep disturbance, and altered appetite among many others.^35^ Chronic pain due to musculoskeletal (MSK) disorders is a frequent condition affecting almost 20%^14^ of the world's population. In Europe, it was estimated that 19% reported a moderate to severe intensity of pain, many of which did not receive appropriate medical care and almost 40% would receive inadequate treatment.^4^ In Latin America, the prevalence ranges from 16.8% to 40.3%.^28^

In general, there is consensus among clinical experts that treating chronic pain is complex mainly because of the coexistence and interaction of biological, psychological, and social factors. This context explains inadequate responses to monotherapies requiring a comprehensive multimodal and multidisciplinary management. In addition, it has been argued that a lack of knowledge on the physiopathology and management of chronic pain increases the need of trained health professionals with specific skills to treat chronic pain, which ultimately leads to patients being treated inadequately as if they were suffering an episode of acute pain. Moreover, the use of opioids is still influenced and limited by medical, ethical, cultural, and legislative factors that lead to a weak prescription, subdosage, and poor adhesion.^4^ There is also a generalized concern regarding the availability of guidelines for the management of chronic pain. Despite these guidelines exist, there are only few of them available; many are very specific; and it has been observed scarce implementation in routine clinical practice.

Furthermore, chronic pain is usually considered a symptom secondary to another disease, which leads health professionals to be highly focused on the treatment of the primary aetiology. However, in many cases, the unwanted consequences of chronic pain, such as decreased quality of life and disability, depend exclusively on the pain domain and, hence, require management that relies largely on pain-specific measures. In addition, treating and managing the aetiology of certain conditions does not necessarily result in a solution to the problem of chronic pain. For example, although hip replacement is the best treatment for severe hip osteoarthritis (OA) and most patients recover mobility, some remain with chronic pain. Thus, addressing chronic pain as a health problem seems clinically reasonable.

From a public health perspective, chronic pain is a prevalent problem associated with high burden of disease and elevated costs. Several studies have estimated the economic impact of chronic pain from different perspectives and using different approaches and/or methodologies. Some have looked into the financial implications of chronic pain as a general condition, and others have focused on specific prevalent pathologies associated with chronic pain. For example, in the United States, chronic pain costs were estimated higher than those for heart disease, diabetes, or cancer.^20^ Other European countries such as Ireland, Sweden, the United Kingdom, and Germany have also attempted estimating costs with the aim of revealing the magnitude of this health problem. However, it still remains uncertain the economic impact of chronic pain on developing countries where the provision of health services is more limited.

On the side of health consequences of chronic pain, there is more consensus and much more has been described in the literature. More broadly, in terms of burden of illness, the Global Burden of Disease (GBD) study stated that MSK disorders were responsible for more than 120 million years lived with disability (YLDs) and account for more than 21% of the worldwide disability.^13,22^ However, it remains unknown the role of the pain domain on this disability estimate. In a recently published study in Chile, the burden of MSK disorders associated with chronic pain was the first cause of burden.^42^

Chile is an upper-middle-income country that provides limited coverage to manage chronic pain. Furthermore, the incorporation of new services to the coverage scheme is driven by an evidence-based priority setting process, where burden of disease, financial burden on families, and other health consequences are relevant criteria to be considered. To contribute to the priority setting process, we performed a cost–consequence type economic evaluation to characterize health and nonhealth costs due to chronic pain in Chile. More specifically, this study aimed to estimate the expected annual cost as well as the magnitude of depression, anxiety, disability, and productivity loss attributable to pain related to MSK disorders.

## 2. Methods

The expected cost and consequences were estimated using mathematical modelling. We implemented a de novo state transition Markov model to characterize changes in severity over time. We focused on pain related to MSK disorders because of its high prevalence allowing us to capture most of the associated economic and health burden. In addition, given the epidemiological and administrative data available as well as the relevance for public health, we restricted the analysis to 6 MSK diseases. The selection of these pathologies was submitted for consideration and agreed by a panel of experts, consisting of a group of specialists of the areas of anaesthesiology, traumatology, palliative care, rheumatology, and physiotherapy. The selection of the panel of experts was supported by the executive board of the Chilean Association for the Study of Pain (ACHED), the leading local scientific society that gathers a multidisciplinary group of experts with interest in the study and treatment of pain.^1^ The selected pathologies were: (1) OA of the knee; (2) OA of the hip; (3) lower back pain (LBP); (4) chronic shoulder pain (CSP); (5) myofascial syndrome (MFS); and (6) fibromyalgia (FM). Although FM is possibly a disease of the central nervous system and peripheral nervous system, it was considered relevant to the analysis as the pain it produces is expressed as chronic MSK pain. There was consensus among experts that MFS was a relevant and common MSK health problem leading to chronic pain, often misdiagnosed, poorly managed, and that less is known among health care professionals, hence it was considered important for this analysis.

### 2.1. Mathematical model

**Figure 1.**
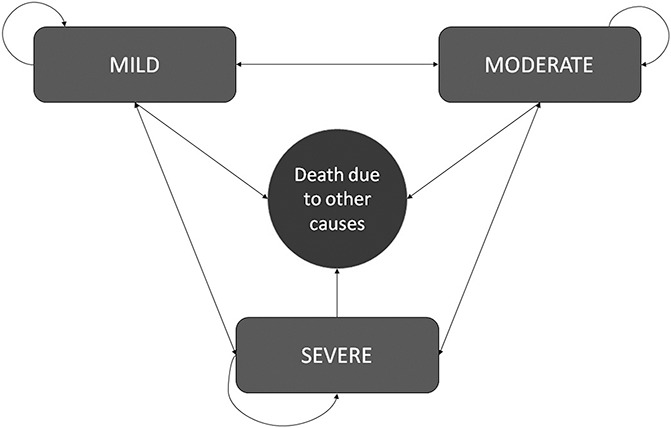
Markov model to represent chronic pain. The figure represents the natural history of chronic pain where patients transit through a 4-state Markov model. The 4 health states that make up the model are based on the intensity of pain: mild, moderate, severe, and death from other causes. Patient's transit through the model irrespective of the pathology of origin based on the transition probabilities obtained through an expert elicitation exercise.

To estimate the costs and consequences, a mathematical model was built in Excel (Fig. 1) to represent the natural history of chronic pain. To differentiate the different possible health states within chronic pain, we implemented the most common way that the patients are assessed based on the severity/intensity of pain through the visual analogue scale (VAS).^15^ Hence, the model was implemented defining 4 health states: (1) mild chronic pain, (2) moderate chronic pain, (3) severe chronic pain, and (4) death from other causes. The model assumes that patients do not die because of this cause (chronic pain), meaning that health loss is given mainly from a decrease in quality of life. It also assumes that patients transit between Markov states independently of their aetiologies. The structure of the model and assumptions were discussed in an advisory meeting where the panel of experts validated the model.

For the purposes of this study, chronic pain was defined as an unceasing pain lasting more than 3 months or a pain that persists beyond the normal healing time, which usually has no protective function, impairs health, and causes disability in patients who suffer it.^18^ Health states were defined based on intensity revealed by the VAS^15^:

1. Mild chronic pain (VAS 1–3): Quality of life of patients is not affected. They can perform daily living activities without difficulty. Functionality and mobility is preserved, and work productivity is not affected.
2. Moderate chronic pain (VAS 4–6): Quality of life of patients as well as work productivity is affected mild to moderately. In this category, the perception of patients becomes more important, and it is expected that those who perceive pain see their quality of life and work productivity affected moderately. Therefore, they are expected to consult more than those who perceive mild pain.
3. Severe chronic pain (VAS 7–10): Quality of life and work productivity are severely affected. Mobility and functional capacity are impaired, and self-care activities cannot be performed or with difficulty.

The cohort of patients with chronic pain entered into the model to one of the 3 possible abovementioned health states. The initial allocation of patients to each health state by pathology (except MFS) in the model was obtained from the 2010 National Health Survey (NHS) based on the proportion of patients who reported intensity of chronic pain.^19,25^ Because MFS was not assessed in the NHS, the initial distribution of patients (at time 0) was assigned based on expert consensus. This approach was preferred to an international estimate because it was more feasible to be accepted for local policy-making. It was suggested that between 5% and 10% of patients (average 7.5%) see a specialist when the intensity of pain is classified as mild, 35% consult when moderate, and 60% when severe. After entering the model, patients could transit between health states according to transition probabilities. This was considered a reasonable approach, as it is expected that patients with chronic pain, depending on the current management, will see their health status improved or worsened as reflected by the intensity of pain. For example, a patient suffering from mild chronic pain may progress to moderate, severe, remain mild, or die from other causes (Fig. 1). This transition occurs assuming the usual/current pharmacological and nonpharmacological management that an average Chilean patient receives. A 1-month length cycle was modelled, as it was considered a reasonable period to characterize significant changes. It should also be noted that transition probabilities remain constant over time and are not dependant on the aetiology originating chronic pain.

The Chilean cohort of patients was estimated based on the reported prevalence of each MSK disorder obtained from the NHS^25^ (Table 1). It was assumed that all prevalent cases have an actual diagnose, although it is expected that a proportion of those suffering mild and moderate chronic pain will not consult. A sensitivity analysis was performed exploring the impact on the results when 50% of patients with mild and 25% of patients with moderate chronic pain have not been diagnosed.

**Table 1:**
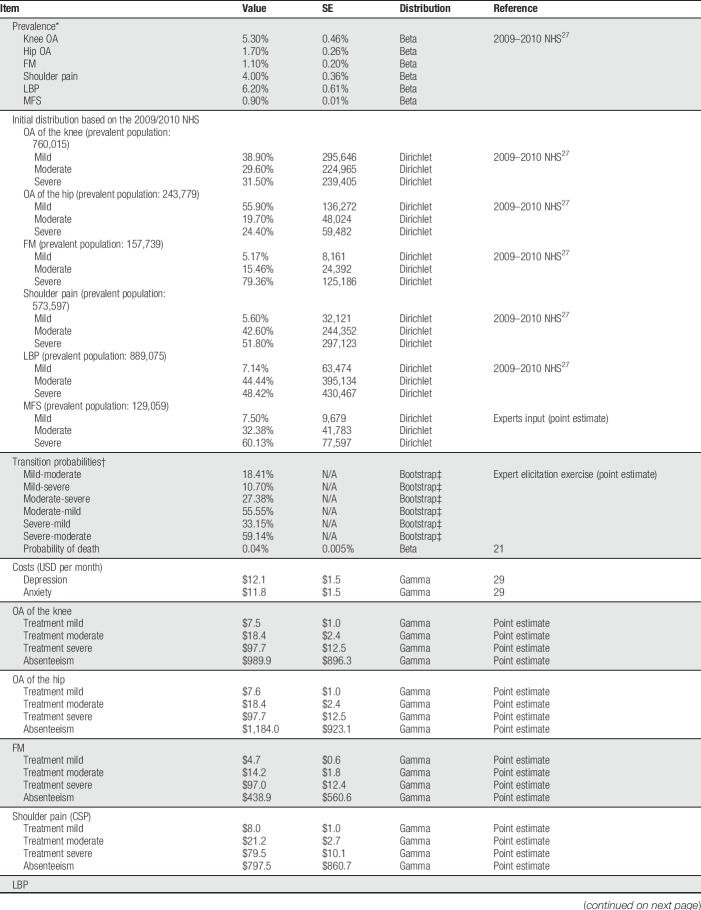

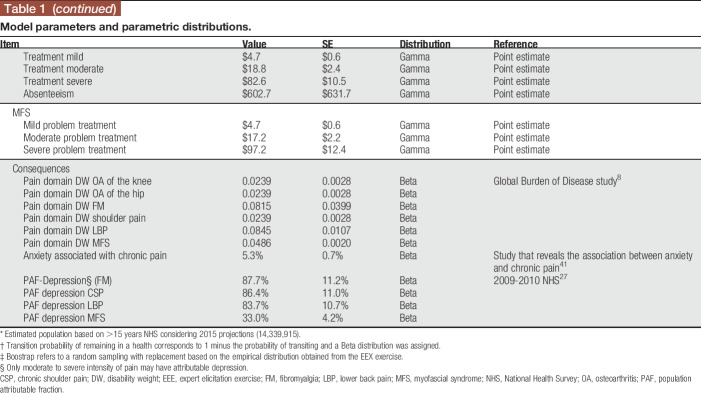
Model parameters and parametric distributions.

### 2.2. Transition probabilities

Given the lack of data in the published literature to estimate transition probabilities for this model, they were obtained through an expert elicitation exercise as suggested by Soares et al.^33^ This methodology allows not only to obtain the expected probability value but also the underlying uncertainty revealed by experts. Hence, the average probability was accompanied by an empirical probability density distribution built from a 21 × 21-squared matrix. These probabilities were estimated based on a 6-question survey completed by a total of 13 clinical experts (anaesthesiologist, traumatologists, and rheumatologists). Each expected probability corresponds to the weighted average obtained from the joint distribution of all experts considering the total of available options per question (21 × 13 = 273 per question). Finally, the probability of death was obtained from the mortality series reported by the Department of Epidemiology at the Ministry of Health.^26^ The annual mortality (5.1 per 1000 inhabitants) was converted to a monthly mortality (0.42 per 1000 population) to fit the model assuming that death occurs at a constant instantaneous rate. This method allows to estimate a Bayesian credibility interval that is largely more appropriate to make inference and more intuitive for decision-making.

### 2.3. Consequences

A literature review was conducted to identify the most relevant consequences of chronic pain. The list of consequences summarized in Table 2 was then assessed by the panel of experts based on available data and their impact on health. At last, 4 consequences were considered in the analysis: disability, depression, anxiety, and productivity loss. Disability was measured as YLDs assuming that there is no mortality attributed to pain. Years lived with disability represent the equivalent number of years spent with disability that are estimated using the incidence, disease duration (in years), and corresponding disability weight. The latter ranges from 0 to 1 and reflects the severity of the disease, where 0 represents perfect health and 1 represents disability equivalent to death.^36^ The disability weights were obtained from the GBD study^32^ and adjusted to represent the pain domain of disability. Depression and anxiety were measured as prevalence of patients where the cause was attributed to chronic pain. For depression, it was possible to calculate the population attributable fraction (PAF), which corresponds to the proportion of cases of a disease that can be avoided in a given population if a given risk factor was not present.^39^ For this purpose, data from the 2010 NHS were used to estimate this parameter for all diseases except MFS. For the latter, a literature review was conducted to obtain the proportion of patients who had moderate or severe pain and also had depression (not necessarily attributed to pain).^41^ It was also assumed that patients with mild pain could suffer from depression associated but not attributable to chronic pain.^12^ This was considered reasonable, as most mild chronic pain cases may develop depression due to other causes other than pain. For anxiety, a literature review was conducted to estimate the proportion of patients in the whole population with anxiety and chronic pain.^41^

**Table 2:**
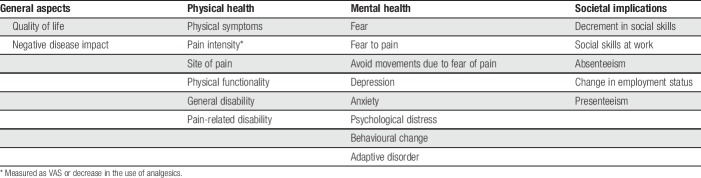
Health and non-health consequences associated with chronic pain.

**Figure 2.**
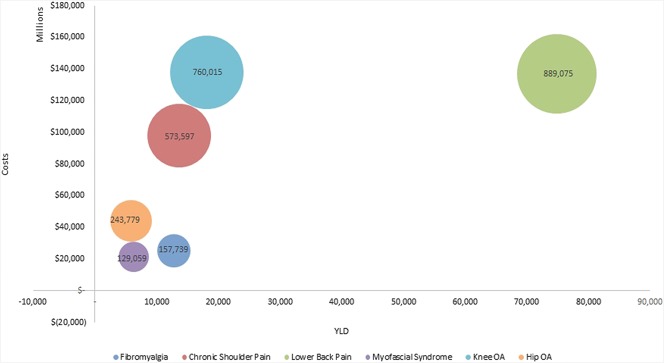
Schematic representation of costs and consequences of chronic pain. The figure represents the structure of this cost–consequence study. It identifies the cost components as well as the health and nonhealth consequences of chronic pain because of MSK disorders. MSK, musculoskeletal; OA, osteoarthritis; YLD, year lived with disability.

Finally, productivity losses were measured as monetary costs following the human capital approach. This method is based on the association between productive time and health.^9^ Thus, absenteeism can be perceived as a loss of investment that a society incurs because an individual has less productive capacity. This methodology suggests that a good proxy to productivity loss is the average market wage. We analysed the 2014 to 2015 private sector medical leave database to estimate the total number of medical leaves per MSK disease. This database includes all possible diagnoses according to the International Classification of diseases 10th version (*ICD-10*) of the World Health Organization.^40^ The use of this database implied the assumption that patients treated in the private sector (approx. 18% of the Chilean population) are equivalent to that of the public sector (approx. 82% of the Chilean population). We are aware that this estimate might be a source of underestimation because these MSK problems are more prevalent in population insured in the public sector. Although more prevalence does not necessarily mean more absenteeism, we did not have access to data from the public sector. We adopted a societal perspective; hence, all costs were measured, including those bared by the patient. Figure 2 summarizes the consequence structure as assumed in the model. Data were analysed using STATA 13.

### 2.4. Costs

Costs were identified, measured, and valued.^8^ Health resources were identified (ie, doctor visits, physiotherapy sessions, type of treatment, etc) and measured (quantity and frequency of use of each health resource) from different sources including international and local clinical guidelines, expert estimates as well as the cost study^27^ performed by the Ministry of Health every 3 years to estimate the annual premium per beneficiary in the context of the universal coverage health benefit plan (GES). The latter source was most relevant to estimate the cost of OA of the knee and hip, as they are financed through the GES plan and have associated specific health resources for the general management and treatment. All these sources are recommended in the economic evaluation guideline elaborated by the Ministry of Health.^24^ To put a monetary value to each health resource, “the published public payer (FONASA) annual tariff^10^ and the 2011 Health Resources Cost Study” requested by FONASA^6^ were used. For pharmaceuticals not included in any of these data sources, the average price was requested to the institution responsible for the purchasing of national health supplies, which provided the average price for 2015. This process allowed for the construction of baskets of health resources that were built for all 6 MSK disorders as well as for each consequence (depression, anxiety, and disability), which were then validated by experts.

It was assumed that all the clinical management of each MSK disease was intended for pain relief and hence, was a good proxy to quantify the impact of disability related to pain as a consequence. The costing of each MSK disease required the identification and gathering of health resources according to the 4-health state model structure. Four main cost categories were identified: (1) medical visits, (2) pharmacological treatment, (3) physiotherapy, and (4) hospitalization. It was assumed that only a proportion of patients with severe chronic pain may require hospitalization for this purpose. The most common reason is exacerbations of chronic pain, which are generally severe, disabling, with no specific related cause, and generally requiring multiple admissions.^23,38^ The usual pharmacologic treatment used on an outpatient basis by patients in Chile was obtained from a previously validated and published population-representative telephone survey.^29^ This information was complemented and validated by experts.

Finally, the same database and assumptions were used to estimate productivity loss as cost. In this case, only the costs bared by the health system were considered; therefore, we calculated only the proportion of days payed by the health system. In most cases, it does not correspond to 100% of the approved number of days established in the medical leave.

All costs were measured in 2015 Chilean pesos and converted to US dollars (USD) using 2015 purchasing power parities (1 USD = 394.35 Chilean pesos).^30^

Years lived with disability correspond to the equivalent number of years spent with maximum disability (equivalent to death). This metric requires incidence data, disease duration in years, and a disability weight, which reflects the severity of the disease where 0 represents perfect health and 1 represents disability equivalent to death.^36^ The disability weights were obtained from the GBD study^32^ and adjusted to represent the pain domain of disability. Two other relevant consequences identified corresponded to mental disorders, depression, and anxiety. The aim was to estimate the prevalence of patients with depression and/or anxiety with selected MSK disorders where the cause was attributed to chronic pain. For depression, it was possible to calculate the PAF, which corresponds to the proportion of cases of a disease that can be avoided in a given population if a given risk factor was not present.^39^ Data from the 2010 NHS were used to estimate this parameter for all diseases except MFS. For the latter, a literature review was conducted to obtain the proportion of patients who had moderate or severe pain and also had depression.^41^ It was also assumed that patients with mild pain do not have depression attributable to chronic pain. In addition, patients with moderate or severe chronic pain can develop mild or moderate depression (not major). A literature review was also conducted to estimate the proportion of patients in the whole population with anxiety and chronic pain.^41^ Finally, productivity losses were measured as monetary costs following the human capital approach. This method is based on the fact that the association between productive time and health^9^ is the average market wage. The 2014 to 2015 private sector medical leave database was examined to estimate the total number of medical leaves according to the International Classification of diseases 10th version (*ICD-10*) of the World Health Organization.^40^ It was assumed that medical leaves in the private sector (approx. 18% of the Chilean population) are equivalent to that of the public sector (FONASA approx. 82% of the Chilean population), given we had no access to the latter. Data were analysed using STATA 13.

### 2.5. Sensitivity analysis

A 1-way deterministic sensitivity analysis was performed to determine the effect of variations of one parameter on the results, ceteris paribus. All model parameters were tested through a 1-way sensitivity analysis. Furthermore, to characterize second-order uncertainty, a probabilistic sensitivity analysis was performed using Monte Carlo simulations (5000 iterations). Parametric distributions were assigned to all parameters accordingly (Table 1).

## 3. Results

The expected costs per month for the therapeutic management of mild, moderate, and severe chronic pain per patient were USD $63.5, USD $101.82, and USD $734.5, respectively. The higher cost of severe chronic pain is mainly explained by the high probability (90%) that patients require an emergency department visit while suffering severe chronic pain, which, in some cases, also leads to hospitalization.

Table 3 report the point estimate and its 95% Bayesian credibility interval following 5000 Monte Carlo simulations. The results reveal important uncertainty around point estimates, which is reflected through the wide credibility interval. The annual expected cost for the 6 pathologies reached $1387.2 MM (ICB 95% $792.71 MM and $2274.6 MM). This amount corresponds to what the health system (public and private) in Chile annually is expected to incur for the management of these 4 chronic pain consequences. This result assumes that all prevalent cases have an actual diagnose. When assessing a different scenario where 50% of patients with mild and 25% of patients with moderate chronic pain are not diagnosed, the total expected cost drops to $1095.89 MM, thus decreasing by 21%.

**Table 3:**
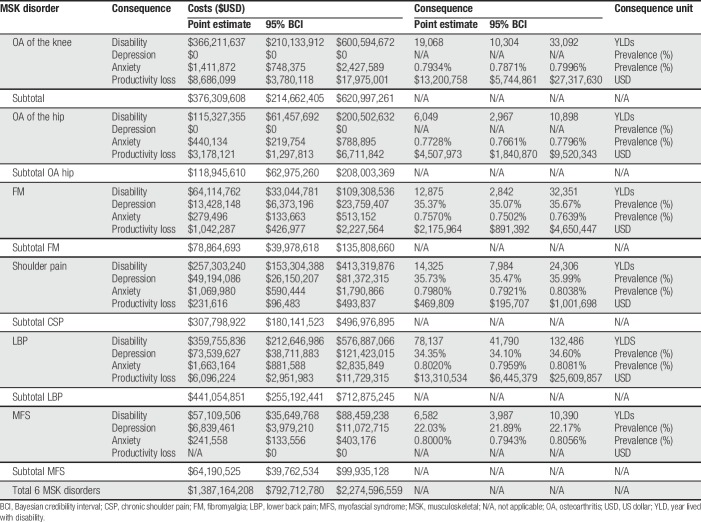
Probabilistic sensitivity analysis: point estimates and 95% BCI for costs and consequences of MSK disorders.

Of the MSK diseases studied, LBP and OA of the knee generate the highest costs, occupying 31.8% and 27.1% of the total expected costs, respectively. For each MSK disease, the main chronic pain cost component was disability. The distribution of this cost comprises mainly of physiotherapy (32.4%) and medical visits (41.7%) followed by hospitalization (21.1%) and medications (4.8%). For example, out of the total estimated cost per pathology, 97.32% of OA of the knee, 96.96% of OA of the hip, and 88.94% of MFS correspond to the therapeutic management of chronic pain, our proxy to disability. A proportion of the total cost is due to chronic pain attributed depression reaching approximately 17% and 16% of FM and CSP, respectively. It should be noted that it was not possible to estimate the cost/consequence associated with the productivity loss due to MFS because it is not identifiable through *ICD-10* code.

In terms of consequences, both, LBP and OA of the knee, were the most disabling conditions associated with 78,137 and 19,068 YLDs, respectively, which were explained only by the pain domain of disability. In total, the pain domain is responsible for 137,037 (ICB 95% from 69,873–243,523). Years lived with disability in patients diagnosed with these 6 MSK diseases. When assessing depression and anxiety, the higher annual costs were driven by LBP and CSP reaching $75.20 MM and $50.26 MM with a prevalence of 2.06% and 1.38%, respectively. Notably, because the PAF of OA knee and hip were assumed zero, no cases of depression attributable to chronic pain were estimated. Similarly, the estimated anxiety costs are correlated with the prevalent population.

An important consequence of chronic pain is given by the loss of productivity. The magnitude of this consequence is determined by the prevalent population and the average number of days of medical leave associated with each diagnose. Lower back pain and OA of the knee produce the largest productivity losses accounting for USD $13.31 MM and USD $4.51 MM per year, respectively. Out of these, only a proportion is incurred by the health system (the cost of productivity loss).

## 4. Discussion

Chronic pain is a common public health problem that causes considerable disability, impaired quality of life, and several other undesired negative consequences. The way that most health systems have addressed this public health problem is mainly through the implementation of strategies focused on the clinical management of the primary aetiology lacking a more comprehensive treatment with a focus on chronic pain. This study produced an estimate of the expected annual cost and a set of consequences attributable to chronic pain, which aims to contribute to the characterization of chronic pain as a public health problem. This is, to the best of our knowledge, the first study of its kind in Latin America.

Lower back pain and OA of the knee were the 2 most costly diseases occupying 31.84% and 26.88% of the total expected costs, respectively. The cost attributed to the management of the disease, our proxy to disability, accounted for most of the total cost. In terms of YLDs, the role of the pain domain was critical leading to 131,559 YLDs in patients diagnosed with these 6 MSK diseases. As a reference, in Chile, the last nationwide burden of disease study (2008), which did not include low back pain or MFS, revealed that the first cause of burden was hypertensive disease that accounted for 257,814 disability-adjusted life years. Thus, only the pain domain of these 6 MSK diseases explains more than half of the disability associated with the main cause of burden in Chile. This can be complemented with recent published data showing that YLDs are lost mostly during productive ages for many MSK diseases.^42^

The results of this study differ from some reported in the literature where an important component of the total cost relied on productivity loses.^7,17^ On average, costs due to productivity losses accounted for 4% only. This can be partly explained by the fact that we could assess reported medical leaves for the private sector, which comprises only 18% of the total population. Hence, assumptions had to be made regarding the remaining 82%, which probably underestimates the total expected costs. In fact, out of the total medical leaves during 2015, 57% corresponded to the public sector and 43% to the private sector. Further analysis is required on availability of data from the public sector.

In terms of total direct cost of chronic pain in Chile relative to the GDP, 0.417% is spent on chronic pain due to MSK, which is smaller than what has been published elsewhere.^5,7^ Several reasons may explain this difference. The health care structure and relative low cost of human resources within the health care system compared with Europe may be one reason to explain the differences in the results. Nevertheless, the differences may be also explained because of the lack of information on medical leaves from the public sector, and numerous diseases causing chronic pain that were not within the scope of the analysis.

Published studies used different methodologies to estimate the cost of chronic pain. The most frequent was the use of a top-down approach of patients or national health surveys to capture the incremental cost of chronic pain compared with patients diagnosed with the same basal disease but not suffering chronic pain.^5,7,16^ Although methodological differences limit the comparability of results, other studies have found similar results in terms of public health expenditure of chronic pain or associated diseases in Europe. For example, the economic impact of chronic pain in Portugal showed that costs reached 2.71% of annual GDP.^2^ Similarly, in 2012 in Ireland, the economic impact in terms of GDP reached 2.86%.^31^ Evidence from Sweden, while also revealing a high economic impact, they also highlighted the relevance of productivity loses in those unable to work because of pain or early retirement.^11^ More specifically, the United Kingdom and Germany estimated the overall cost of back pain which alone reached 1.5% and 2.2% of annual GDP, respectively.^21,37^ As mentioned before, we did not identify published evidence in Chile or Latin America reporting the costs and consequences of chronic pain using similar methodologies.

In addition, there are 2 possible consequences that were not measured: early retirement and presenteeism. Although the former corresponds to the retirement before the legal age because of the disability related to chronic pain, the latter refers to the decrements in productivity because of a health problem in workers who continue attending their jobs. These consequences have a direct impact on patients and society. However, in Chile, the costs associated with these 2 problems do not fall in the health system budget; therefore, excluding them is consistent with our analysis that adopts the health system perspective.

One important limitation regarding productivity costs and consequences is that our estimates do not capture nor characterize medical leaves of patients diagnosed with MFS. Unfortunately, to date, this important health problem has not been classified by the *ICD-10*, and therefore, it cannot be identified in the corresponding database. However, as it corresponds to a very specific diagnose and physicians are still not trained correctly to identify it, part of it could be possibly assumed as other diagnoses such as FM. The magnitude of this is unknown, and it is not possible to determine the magnitude based on available data. A possible solution relies on the still ongoing development of the new classification for chronic pain to be included in the upcoming 11th revision of the ICD.^34^ This will allow us to study chronic pain as a whole and enhance the development of guidelines to support a specialized chronic pain treatment.

Another limitation is that our study does not consider all MSK diseases causing chronic pain. For example, epicondylitis and cervical pain are 2 prevalent diagnoses that were not evaluated but have also shown to cause significant costs and consequences attributed to chronic pain. On the other hand, when considering mental health problems, adjustment disorder is one of the most commonly described in these patients, and it is probably attributed to chronic pain. However, because of the difficulty in obtaining the required data, it was not possible to consider this important result in our analysis.

One general limitation of our study was the availability of data, hence the use of assumptions and expert opinion to be able to conduct the evaluation, which we acknowledge is not considered high-level evidence. However, it should be noted that there is an increasing consensus that mathematical modelling is an adequate instrument to estimate costs and outcomes. We argue that our structural assumptions (mainly model structure and transition probabilities) are reasonable because they are consistent with local reality, the parsimony principle that facilitates knowledge translation, and accepted by local experts. In addition, we believe that when evidence aims to support decision-making, this should not be postponed because of lack of evidence; instead, we believe that the best available evidence should be pursued. As an example to this aim, expert elicitation methods were developed, tested, and have been broadly discussed in the field of health economics.

Despite the limitations described above, this study is the first to show some of the main consequences and associated costs of chronic pain in Chile, and to the best of our knowledge, one of the few studies specifically designed to characterize costs and consequences. These results confirm what has been widely described in international literature, ie, chronic pain is a common health problem causing severe disability and impacting quality of life from many dimensions. This is especially interesting in Chile, where many of the conditions included in this study can be managed with health resources already included in the Chilean health benefit plan. This suggests that the current practice is not being sufficient to decrease the magnitude of this problem. We hypothesize that this occurs because patients are being managed focusing on the etiology of the disease, and pain is addressed as a secondary symptom that is usually treated as if was an acute episode of pain.

Future research needs to be performed to assess the effectiveness of health programs to address chronic pain as the main health problem. This considers clinical interventions but also other more innovative strategies such as training patients how to live with pain. The optimization of existing primary and secondary care before implementing new health interventions such as specialized pain centers, education of health professional to offer better treatments in primary care, and the generation of clinical guidelines are some examples of these type of strategies. If effective programs can be implemented, the measurement of their impact will benefit from this study as a baseline characterization of the problem.

Finally, the value of this piece of research is given partially by the reported magnitudes but also by the methodological approach used to produce this information; it provides a good alternative to estimate legitimate results that are locally valid, accepted, and could be used for decision-making.

## 5. Conclusion

There are important health and nonhealth consequences associated with chronic pain, which are costly to health systems and society as a whole and they impact negatively in an important percentage of the population. More attention needs to be given to this health problem not only because it is costly but also because adequate pain treatment should be considered a human right, and it is the health system's responsibility to provide it.^3,14^

## Disclosures

M.A. Espinoza, C. Vargas, C. Balmaceda, and R. Rojas have participated in the execution of this study under the contractual terms as academics of Pontificia Universidad Católica (PUC). P. Zitko and M.F. Rodríguez have received fees from PUC. N. Bilbeny, M. Ahumada, and M.E. Eberhard are members of the ACHED, have not received fees for their participation in this study, and do not have competing interests.

This study was funded by the Chilean Association of Study of Pain (ACHED). We are aware that part of this funding was obtained through an unrestricted grant provided by Grunenthal to ACHED. The study was performed under strict contractual clauses of independence regarding the execution of this study including: study design, data collection, interpretation of data, analysis of the results, and elaboration of the manuscript.
